# *R*adioresistant, *R*are, *R*ecurrent, and *R*adioinduced: 4*R*s of Hadrontherapy for Patients Selections

**DOI:** 10.1016/j.ijpt.2024.100737

**Published:** 2024-12-31

**Authors:** Barbara Vischioni, Amelia Barcellini, Giuseppe Magro, Marco Rotondi, Marco Durante, Angelica Facoetti, Juliette Thariat, Ester Orlandi

**Affiliations:** 1 Department of Internal Medicine and Therapeutics, University of Pavia, Pavia, Italy; 2 Department of Clinical, Surgical, Diagnostic, and Pediatric Sciences, University of Pavia, Pavia, Italy; 3 Radiation Oncology Unit, Clinical Department, CNAO National Center for Oncological Hadrontherapy, Pavia, Italy; 4 Medical Physics Unit, Clinical Department, CNAO National Center for Oncological Hadrontherapy, Pavia, Italy; 5 Radiobiology Unit, Research and Development Department, CNAO National Center for Oncological Hadrontherapy, Pavia, Italy; 6 Département de Radiothérapie, Centre François Baclesse, Caen, France; 7 GSI Helmholtzzentrum für Schwerionenforschung, Biophysics Department, Darmstadt, Germany; 8 Department of Physics, Institute of Condensed Matter Physics, Technische Universität Darmstadt, Darmstadt, Germany; 9 Department of Physics "Ettore Pancini," University Federico II, Naples

**Keywords:** Hadrontherapy, Rare tumor, Radioresistant tumor, Recurrent tumor, Radio-induced secondary tumor

## Abstract

**Purpose:**

To describe the role of hadrontherapy (HT) in treating radioresistant, rare, recurrent, and radio-induced tumors, which can be defined, in assonance with the 4Rs of radiobiology, the “4Rs” of HT indications.

**Materials and Methods:**

This is a narrative review written by a multidisciplinary team consisting of radiation oncologists, radiobiologists, and physicists on the current literature on HT, particularly carbon ion radiation therapy. To refine HT indications within the context of the “4Rs” framework, we evaluated tumor histologies across different clinical indication settings and emphasized the radiobiological mechanisms contributing to the effectiveness of HT.

**Results:**

For rare, radioresistant, recurrent, and radio-induced tumors, HT has proven to be effective and safe, achieving high rates of local response with mild toxicity. The current review shows that the biological parameters can assist clinicians in identifying appropriate cases for HT treatment.

**Conclusion:**

Biological characteristics of the tumor support the administration of HT in radioresistant, rare, recurrent, and radio-induced tumors and should be considered during multidisciplinary discussions.

## Introduction

Hadrontherapy (HT), also known as particle radiation therapy (RT), is rapidly attracting increasing attention in the medical community due to growing clinical data demonstrating benefits for cancer patients compared to conventional x-ray RT (X-RT). HT advantageous physical and dosimetric properties allow for precise dose sculpting to the tumor while sparing surrounding normal tissues, thanks to the deep dose gradient beyond the tumor target.^1^

The vast majority of HT treatments use protons (proton beam RT [PBT]), but 14 centers are using carbon ions, which add to the physical advantages of the protons a number of unique biological properties, such as increased relative biological effectiveness (RBE) and reduced oxygen enhancement ration.^2^ However, protocols for the differential use of specific particles in clinical settings (PBT vs carbon ion radiation therapy [CIRT]) are not yet well-defined, and the level of evidence supporting differential indications of PBT/CIRT versus X-RT in cancer guidelines remains limited.^3^, ^4^, ^5^

Generally, tumors in challenging locations (close to sensitive organs at risk), with irregular shapes, or those requiring reirradiation after previous radiation courses, may be good candidates for HT. This is due to the HT physical properties, which offer potential benefits in reducing treatment-related toxicities. Clinical evidence of this reduced toxicity is now established in several clinical trials.^6^, ^7^, ^8^, ^9^ The biological properties of CIRT, as reported in preclinical research^10^ and, by the large Japanese experience in clinical treatments,^11^ make it particularly suitable for radioresistant and hypoxic tumors.

Radioresistant tumors are often rare entities, which partly explains the difficulties in conducting clinical trials and accumulating sufficient evidence for the differential use of CIRT versus X-RT. Additionally, HT is often used for recurrent and radio-induced tumors, considering the poor outcomes of conventional treatments, including X-RT. It has been hypothesized that recurrence following RT is driven by reactivated persistent or progenitor cancer stem cells,^12^, ^13^, ^14^ exhibits strong hypoxia hallmarks,^15^ and is prone to overcome intrinsic cellular damage repair mechanisms. Recent advances in translational and molecular research have reinforced the lessons learned from the classical 4Rs of radiobiology in patients, highlighting the relevance of cellular mechanisms that determine the success or failure of standard clinical X-RT: *R*-epair of DNA damage, *R*-edistribution of cells during the cell cycle, *R*-epopulation, and *R*-eoxygenation of hypoxic tumor cells.^16^ Moreover, two more Rs have been added: intrinsic *R*-adiosensitivity and *R*-eactivation of immune response.^17^ CIRT is potentially superior to X-RT in all of these Rs. Repair is complicated by the complexity of DNA damage, redistribution in the cell cycle and reoxygenation are less important for the tumor, intrinsic radioresistance is reduced, and preclinical evidence also points to a stronger activation of the immune response.^10^, ^18^ However, whether these laboratory experiments lead to real clinical benefits remains to be demonstrated.

**Figure 1:**
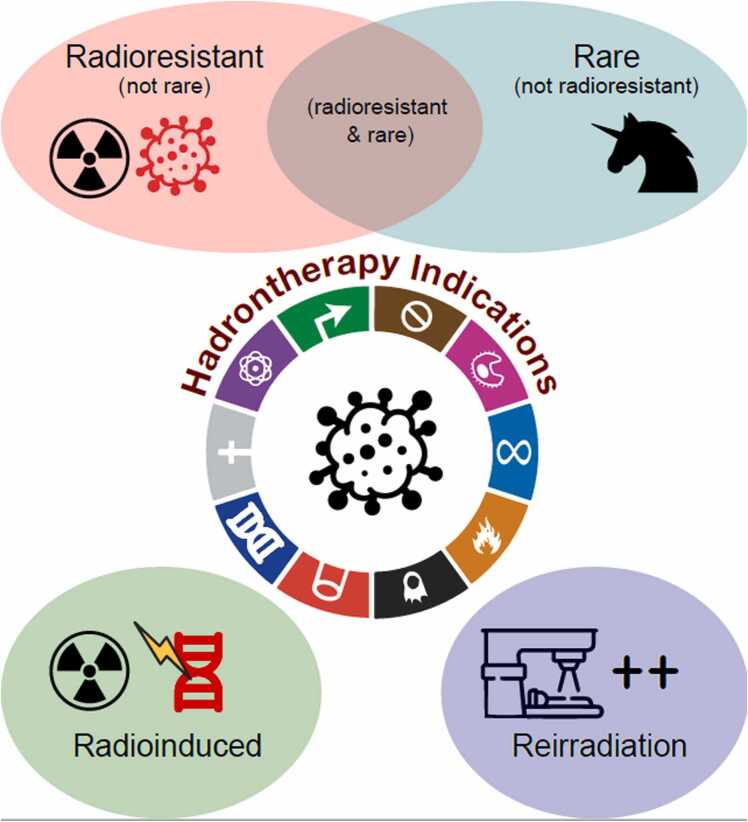
The 4Rs of hadrontherapy indications: R-adioresistant, R-are, R-ecurrent, and R-adioinduced tumors. Figure made by iInkscape Project (https://inkscape.org), taken from Wikimedia Commons public domain (https://commons.m.wikimedia.org/wiki/File:Next-generation_Hallmarks_of_Cancer_wheel_with_labels.svg#filelinks), inspired and adapted from Hanahan and Weinberg.^19^

Based on the preclinical experience, we believe that CIRT can be particularly effective in treating *R*-adioresistant, *R*-are, *R*-ecurrent, and *R*-adioinduced tumors, collectively referred to as "the 4Rs of HT indications" (Figure 1). Within this frame, this article aims to better characterize the indications of HT (and in particular of CIRT) in these “4R” settings by ranking different cancer histotypes according to the different indication settings and highlighting the main radiobiological mechanisms behind the effectiveness of CIRT for rare, radioresistant, recurrent, and radio-induced tumors.

## *R*are and *r*adioresistant tumors

**Figure 2:**
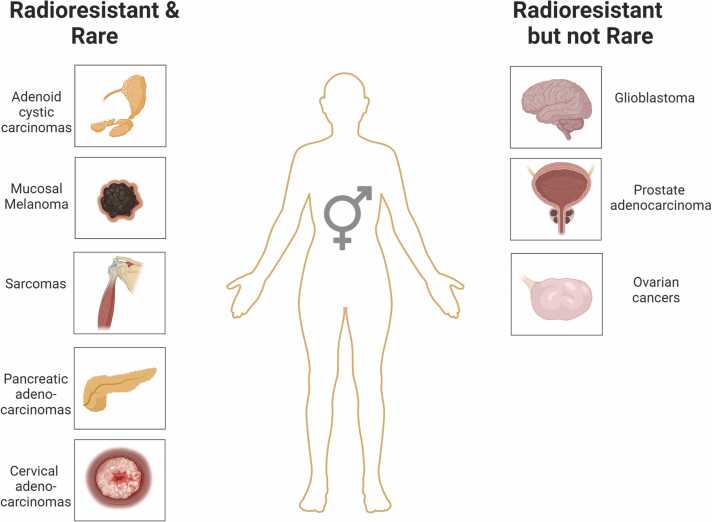
The panel schematically shows “rare and radioresistant” tumors and “radioresistant but not rare” tumors in which HT may potentially be used. Figure made by BioRender.com.

Radioresistant tumors are defined as malignancies that poorly or do not respond to conventional X-RT for intrinsic characteristics, such as hypoxia, low cell proliferating fractions, and classical tumor escape hallmarks. Both genetic radiosensitivity and the tumor microenvironment (TME) can play a role in the clinical definition of radioresistance.^20^ Tumor entities are defined as “rare” if they occur in <6 per 100 000 people each year.^21^ Sometimes the concept of “radioresistant tumor” overlaps with the “rare,” considering that, in the clinical context, most radioresistant histologies are also uncommon. Moreover, due to the rarity of the disease, their precise radiosensitivity/resistance profiles are poorly understood and indagated. It is unclear how radiation should be used, even if increasing evidence justifies the role of HT. Among the rare and radioresistant tumor entities, herein we focus on the most challenging: adenoid cystic carcinomas (ACCs), mucosal malignant melanomas, sarcomas, cervical adenocarcinomas, and pancreatic adenocarcinomas, where CIRT was tested and proved feasible and effective over the traditional therapeutic approaches (Figure 2).

### Adenoid cystic carcinomas

ACC is a very uncommon tumor representing approximately 10% of all malignant salivary gland histotypes. Thus far, no preclinical or in vitro research has systematically examined the effects of CIRT on ACC cell lines. However, biological characteristics support the use of CIRT in ACC treatment. Concerning this, de Mendoça et al^22^ found that, in comparison to normal salivary glands, ACCs had higher levels of the hypoxia-inducible factor (HIF) 1α, which is produced when there is insufficient tissue oxygenation. According to other authors, hypoxia enhanced the characteristics of cancer stem cells and the ACC epithelial-mesenchymal transition.^23^ A lot of studies have indicated that ACC may benefit from CIRT, either as a standalone treatment or when combined with intensity-modulated radiation therapy (IMRT) using photons, in terms of local control (LC), overall survival, and toxicity.^24^, ^25^, ^26^, ^27^ These investigations have examined the efficacy of CIRT in various scenarios, including R2 resections and in cases where surgical intervention is not feasible. Specifically, a study by Akbaba et al^28^ focusing on ACCs of the paranasal sinuses found a higher incidence of toxicity (>G3) following postoperative IMRT combined with CIRT compared to cases where IMRT and CIRT were administered as primary approach. However, no significant differences have been observed in terms of LC. A phase II study is currently ongoing at the Italian National Center for Oncological Hadrontherapy to test the safety and efficacy of CIRT administered through simultaneous integrated boost (SIB) in patients with unresectable or postoperative macroscopic ACC (NCT05733910). CIRT was investigated also in pelvic and gynecological ACCs.^29^

### Mucosal malignant melanomas

Among melanomas, mucosal malignant melanomas are the rarest and the most radioresistant histologies with a dismal prognosis due to the high rates of early metastatic propensity.^30^ Several preclinical studies have shown the advantages of CIRT in overcoming the radioresistance of this histology,^31^, ^32^, ^33^, ^34^, ^35^ and this was the background to test the efficacy of CIRT in this setting. In particular, literature data offer promising results concerning CIRT for head and neck and gynecological melanomas. Ronchi et al^36^ recently reported a retrospective series of 40 head and neck mucosal melanomas treated with a total dose of 65.6 to 68.8 Gy RBE in 16 fractions, achieving a 2-year local relapse free survival of 84.5% superior to the proton beam cohort.^37^, ^38^ Moreover, the CIRT experience on gynecological melanomas is encouraging compared to the historical surgical cohort with an LC between 71% and 86% at 2 years.^39^, ^40^ It should be stressed that the toxicity rates of CIRT in both districts were mild compared to the consequences of a mutilating demolitive surgery, often an option in these challenging cases. Considering the need to improve not only the LC but also the progression and overall survival, CIRT was also evaluated in combination with immunotherapy. Indeed, immunotherapy has dramatically improved the survival rates in melanomas, also in the metastatic setting,^41^ and it is widely accepted that the combination with RT can improve the survival in some solid cancers. In this regard, the ballistic properties of CIRT allow sparing more efficiently the immune cells necessary to trigger an adequate antitumor immune response. Moreover, it has been shown that CIRT might cause more often immunogenic cell death with significant release of inflammation mediators and activation of immunological pathways.^18^ Waiting for the results of an ongoing clinical trial in the recruitment phase aimed at evaluating the effectiveness in terms of LC and PFS of the combination of immune checkpoint inhibitors (ICIs) and CIRT in improving the PFS,^42^ several preclinical data support the hypothesis that CIRT is better than X-RT in fueling the anticancer immune response.^43^

### Uterine cervical adenocarcinomas

Under the umbrella of uterine cervical adenocarcinomas, there are a series of unusual malignancies with a poorer prognosis than more frequent histologies. Considering all the subtypes, cervical adenocarcinomas account for 10% to 25% of all cervical cancer cases but show a growing incidence within the last 20 years.^44^ Due to the lack of data, its clinical characteristics and the best treatment approaches remain largely unclear. Current guidelines for cervical cancer suggest the same strategy for squamous cell carcinoma and nonsquamous subtypes. However, studies on early-stage or locally advanced cervical cancer treated with concomitant chemotherapy and X-RT or neoadjuvant chemotherapy have shown that adenocarcinomas respond locally worse than squamous cell carcinomas.^45^, ^46^, ^47^ Overall, the 5-year LC rates for patients who received conventional X-RT treatment with or without chemotherapy were 36% to 58%.^48^, ^49^ Adenocarcinomas have a higher hypoxic cell fraction compared to the most common histologies, supporting the hypothesis of its intrinsic radioresistance. The radiobiological studies on cervical adenocarcinoma cell lines documented an increased effectiveness of CIRT over X-RT, suggesting that heavy ions can overcome the intrinsic radioresistance of this histology.^50^ These data were confirmed by clinical experiences in which, compared to the historical X-RT cohort, CIRT achieved a better oncologic local outcome, up to 65.2% at 5 years, and concurrent uses of chemotherapy did not affect LC.^51^ Moreover, it has been shown that CIRT is able to upregulate in a dose-dependent manner the expression of PD-L1 both in cervical squamous and adenocarcinoma cell lines, inducing also a clinical PD-L1 expression in both the uterine histologies,^52^ paving the way to future combination with immunotherapy in these difficult-to-cure patients.^53^

### Pancreatic adenocarcinomas

Pancreatic ductal adenocarcinoma is currently a significant worldwide health challenge. It ranks seventh as the leading cause of cancer-related death and will probably overcome breast cancer as the third cause of cancer-related death by 2025.^54^, ^55^ With its increasing incidence and the lack of therapeutic options, its cure might be considered an unmet oncological need. Up to 50% of affected patients are diagnosed at a metastatic stage, 30% with a locally advanced or borderline resectable tumor, and only 20% with resectable disease. Based on the chromosomal rearrangements, there are 4 biological types of pancreatic cancers: stable (20%), scattered (36%), unstable (14%), and locally rearranged (30%), each of them correlated with differences in response to systemic therapy and in clinical outcomes.^56^ The immunosuppressive TME and noticeably greater hypoxia levels compared to the majority of solid tumors are characteristics of pancreatic cancer that might ultimately explain its radioresistance. The thick fibrotic tissue surrounding the malignancy, known as widespread desmoplasia, typical of pancreatic tumor histology, can result in several repercussions, including increased interstitial pressure and blood vessel compression. Moreover, fibrosis and hypoxia might interplay with one another in a complicated connection that strengthens the metastatic phenotype.^15^ X-RT has given so far unsatisfactory oncological outcomes, probably because the dose escalation needed to overcome tumor radioresistance is limited by the close proximity to high radiosensitive normal tissues (ie, bowel, duodenum, and stomach).^57^ The Japanese experience with CIRT in unresectable pancreatic cancer is encouraging with a 2-year LC ranging between 76.1% to 82%^58^, ^59^, ^60^ superior compared to the studies carried out in the Western countries with X-RT,^57^ as predicted also in a tumor control probability model approach.^61^ Considering the dismal prognosis of locally advanced pancreatic cancer and the limited options available, the Japanese results in this tumor are considered the most promising application of CIRT. Further dose escalation is ongoing at the National Institutes for Quantum and Radiological Science and Technology (QST) in Chiba to reach 90 EQD2 Gy.

### Sarcomas

Primary bone sarcomas, like chordomas, chondrosarcomas, and osteosarcomas, exhibit considerable heterogeneity and a low incidence, accounting for <0.2% of malignant tumors.^62^ These tumors are recognized as resistant to conventional X-RT, necessitating higher doses to achieve sufficient LC rates, In vitro data reported that the radioresistance of chordoma cells is associated with the ataxia-telangiectasia mutated (ATM)/ATM and Rad3-related (ATR) pathway, in which RAD51 serves as an important downstream effector.^63^ Furthermore, in in-vitro studies on the human chordoma cell line UCH1-N, high-Linear Energy Transfer (LET) particles, including carbon ions, showed a higher cell-killing effect in comparison to protons.^63^ In chondrosarcoma cells, hypoxia is reported to contribute to radioresistance by stabilizing HIF-1α, activating angiogenesis pathways, and promoting tumor-initiating and invasive tissue mediators.^64^ Hypoxia also promotes cancer stem cell activation, which exhibits self-renewal and resistance, increasing tumor aggressiveness and resistance.^65^ Furthermore, in preclinical studies on multiple chondrosarcoma cell lines, CIRT has shown its effectiveness by inducing prolonged G2 phase arrest and permanent cell damage.^66^ The high levels of HIF-1α contribute to the overall radioresistance observed in human osteosarcoma by activation of autophagy that accelerates the clearance of irradiation-induced DNA-damaging reactive oxygen species.^67^ A recent meta-analysis of 12 studies including 897 bone sarcomas patients who underwent CIRT (526 had chordoma, 255 chondrosarcoma, 112 osteosarcoma, and 4 other sarcomas) reported promising data on LC and overall survival. In particular LC at 1, 2, 3, 4, 5, and 10 years were as follows: 98.5%, 85.8%, 86%, 91.1%, 74.3%, and 64.7%. Similarly, the overall survival rates at 1, 2, 3, 4, 5, and 10 years were: 99.9%, 89.6%, 85%, 92.4%, 72.7%, and 72.1%.^68^ The above-reported proimmunogenic effect of CIRT in inducing abscopal effect, when combined with ICIs, such as anti-PD-1 and anti-CTLA-4, was also described in an LM8 osteosarcoma mouse model.^43^ The authors injected LM8 cells in both the limbs of the mice, but only one of them were irradiated (with X-RT or CIRT) with a concomitant administration of ICI intraperitoneally. The surprising result was the significant reduction of the nontreated limb localizations in the arm CIRT+ICI compared to mice that underwent X-RT+ICI. In addition to the tumors inoculated in the limbs, the model spontaneously formed metastases in the lungs. The authors counted the number of visible lung metastases on the surface of the organs isolated from animals treated with the different regimens and found that CIRT reduced the number of lung metastases more effectively than X-RT, especially when combined with ICIs.

## Radioresistant and not rare

In addition to the rare and radioresistant malignancies, there are also tumor radioresistant but with a high incidence worldwide. They have very different prognoses and heterogenous mechanisms of radioresistance, most of them yet to be characterized. In this regard, future genetic characterization of these tumors may provide valuable insights. The radioresistant phenotype makes them suitable to test the effectiveness of HT, and so far, several trials explored or are exploring the potentiality of PBT or CIRT in this context. Among all the possible histologies currently being treated, we include in this paragraph the most potentially challenging that might pave the way to future HT indications: prostate cancers (PCas), glioblastomas, and ovarian cancers. The rare but not radioresistant malignancies have not been included in the present overview of HT indications, even if HT justification, especially with PBT, might be linked to their location in critical sites (eg, pediatric neoplasms, nasopharyngeal tumors, squamous neoplasms of the female genital tract) and the need for safe dose escalation (Figure 2).

### Prostate cancers

PCa is very frequent and highly curable disease, although it exhibits some form of radioresistance. There is greater radioresistance in the late, early, and quiescence phases. Late-responding tissues have low *α*/*β* values (0.5-6 Gy), with PCa of 3 or lower. For late-responding tissues, fraction size is the dominant factor in determining the radiation-induced effect, with extra doses required to counteract proliferation being far beyond the overall time of the conventional fractionation regimen.^69^ Radioresistance appears to be due to not-proliferating cells in the resting phase and re-entering the cell cycle later. PCa exhibits clonogens with a long kick-off time for accelerated repopulation and fast repopulation by tumorigenic stem cells.^70^ Clinical experiences with high fraction size, that is, hypofractionated and stereotactic X-RT, typically used for low *α*/*β* tumors, have demonstrated good tolerance and tumor control comparable to conventional fractionation so far, with long-term results awaited. Temporal technique-treatment patterns show a decline in the proportions of patients who received conventional fractionation, from 76.0% in 2004 to 36.6% in 2020 (*P* < .001), with moderate hypofractionation increasing from 22.0% to 45.0% and ultrahypofractionation from 2.0% to 18.3%.^71^ In addition to hypofractionation, the physical and radiobiological properties of CIRT could be exploited efficiently in PCa. Japanese series have shown excellent tumor control and no excess risk of toxicities including radiation-induced primaries.^72^ Of note, current data suggests that the MKM model may better predict RBE and outcomes besides further genetic tumor characterization.^73^, ^74^ Considering the Japanese data and the preclinical evidence, the approaches to assess in this field should be: i) the full course of CIRT (from hypofractionation to ultrahypofractionation); ii) the mixed beam treatment, including an early CIRT boost on the prostate and seminal vesicles aimed at increasing the radiosensitivity of the tumors, followed by a pelvic X-RT course to the elective regions.^75^, ^76^, ^77^

### Glioblastomas

Among radioresistant brain tumors, glioblastomas (GBMs) are characterized by a dismal prognosis of about 18 months median survival. Its radioresistance hallmark is dependent on multiple factors such as TME (with its hypoxic features, metabolic alterations, cancer stem cells and heterogeneity), micro-RNAs specific expression, cell cycle deregulation, and alteration of DNA damage repair mechanisms.^78^ To improve RT efficacy in this histology, fraction size and overall treatment time have been reported as crucial. Because GBM cells are quickly proliferating, they redistribute in more sensitive phases after fractions, but at the same time increase in total number of cells to be killed. For this reason, it has been postulated that CIRT might be particularly relevant to face many aspects of radioresistance of GBMs,^79^, ^80^ considering the independence of hypoxia and cell cycle, as well as efficacy on cancer stem cells.^15^ However, because GBMs are poorly anatomically limited within the brain and rapidly growing tumors, it is critical to develop both accurate clinical target volume and adaptive strategies to avoid tumor failures by a geometric miss. Spatially fractionated beams or FLASH CIRT are being investigated intensively and could be implemented in the near future to spare the nontumoral brain.

### Ovarian cancers

In recent years, therapeutic advances in oligo-metastatic, oligo-persistent, and oligo-recurrent ovarian cancers have improved prognosis. The introduction of PARP inhibitors (PARP-i) and anti-angiogenics in this setting has made it essential to find strategies that delay rechallenge with platinum. In this context, RT, traditionally seen as palliative in ovarian cancer, has shifted in its role and is increasingly considered a loco-regional treatment with curative intent, aiming to achieve a long free-drug interval. Even though SBRT had encouraging outcomes for LC, a percentage of women did not receive significant responses in the RT field, and there was a noteworthy rate of progress out of the RT fields (from 79% to 100%).^81^ Indeed, from a radiobiological point of view, BReast CAncer gene (BRCA) wild-type patients and tumors with defective in the homologous recombination DSB repair pathway and/or high genomic instability are more radioresistant to X-RT.^82^, ^83^ On the other hand, BRCA-mutated patients are at risk of higher toxicity when exposed to radiation.^84^ These data should stress the need to tailor RT according both to the molecular background of the tumor and the patient's risk of high toxicity. Clinically, CIRT has proved to be more effective in terms of objective response rate compared to the historical data of SBRT in oligo-metastatic, persistent, or recurrent ovarian cancers, with a mild incidence of adverse events also when combined with radiosensitizing drugs such as PARP-i and anti-angiogenics,^85^ paving the way for further studies necessary to better select patients to treat with X-RT versus particle beam RT.

## Recurrent tumors

Reirradiation presents a significant challenge for radiation oncologists, as it requires ensuring effective tumor coverage, especially since the tumor is likely to be radioresistant after previous treatment. At the same time, it is crucial to protect the surrounding healthy tissues that have already been irradiated. In this challenging setting, literature reported HT advantages in terms of dosimetry and RBE over X-RT for several anatomical scenarios with a mild rate of toxicity.^86^ From a radiobiological point of view, radiation induces an inflammatory reaction in the TME damaging endothelial cells with consequent fibrosis, cyclic hypoxia, and affected cancer-associated fibroblast activity. These alterations are closely related to the revascularization, immunological regulation, and extracellular matrix remodeling phenomena, all of which shape the radioresistant hallmarks of recurring cancers in a previous RT field.^87^ The capabilities to act regardless of the oxygenation status and to trigger the anticancer immune response also affecting the TME^10^ make CIRT theoretically more efficient than PBT or X-RT in the treatment of recurrent malignancies. In addition to their radiobiological hallmarks, the ballistic characteristics of CIRT versus PBT or X-RT allow it to spare the surrounding normal tissues more efficaciously. These proprieties are crucial in the case of reirradiation where the aim of eradicating the tumor is often limited by the intention of keeping an adequate quality of life and relieving patients' symptoms. The reduction of organs at risk’s mean doses might be significantly better with CIRT in comparison to PBT and X-RT^88^ and taken together with sharper lateral penumbra and the higher RBE, justifying its application in the re-irradiation setting. Indeed, both LC and toxicity rates are impressive when we consider CIRT reirradiation at radical doses in scenarios of well-known radioresistant tumor types, such as recurrent pancreatic cancer,^89^ recurrent gynecological malignancies^90^ rectal cancers,^91^, ^92^, ^93^, ^94^ sinonasal,^95^ head and neck tumors (especially salivary gland tumors)^96^, ^97^ and CNS recurrent malignancies.^98^ In this regard, it is of note the high rate of eye preservation (55% at 5 years) in case of reirradiation of recurrent uveal melanoma up to a total dose of 70 Gy RBE. However, sometimes it might be difficult to compare the toxicities of the CIRT series with X-RT since patients indicated for particles usually include patients with comorbidities, lack of alternative treatments, and tumors adjacent to critical organs. Consequently, the observed toxicities reported in the literature may seem more severe for CIRT as compared to those described in the X-RT series for less difficult-to-treat recurrences. In this context, optimizing LET within the target could be a promising strategy in the near future. This would involve applying a lower LET to the normoxic healthy tissue surrounding the tumor, while directing higher LET to the recurrence, which is typically hypoxic and rich in radioresistant clones.

## Radio-induced tumors

A radio-induced tumor is a rare but devastating late postactinic event, difficult to diagnose and manage considering its intrinsic radio- and chemo-resistance. Additionally, it poses a significant challenge for the surgeons because of the previously delivered RT doses. Physical and radiobiological data regarding the risk difference between HT and X-RT in inducing secondary tumors have been summarized by Facoetti et al,^13^ and the reader is referred to that review for a comprehensive understanding of this issue. Hufnagl et al^99^ compared the risk of secondary cancer induction between PBT and CIRT, showing that the most relevant parameters impacting the risk were the position in the field, the dose level, and the RBE value of the target tissue. Nitta et al^100^ reported no statistically significant difference in the incidence of radio-induced tumor in the pelvis (*P* = .388) or out of the pelvis (*P* = .353) in uterine cervical cancer patients treated with X-RT or CIRT.

Table 1 summarizes the main literature evidence about secondary tumors treated with particle beam RT. Overall, 31 patients were treated with HT for a radio-induced tumor arising after a latency time between 5 and 31 years, with a median age at the time of the first treatment of 38 years (range: 3-69 years). Consistent with literature data,^14^, ^101^, ^102^, ^103^, ^104^, ^105^ the histology of radio-induced secondary tumors was rare and aggressive, but compared to sporadic tumors, the survival was worse with even dismal LC. These tumors were mainly treated with CIRT and despite the high delivered doses, the treatment was well tolerated. There was only one case of acute grade 4 toxicity leading to grade 5 late toxicity. This occurred in a patient with a locally advanced nasopharyngeal carcinoma who experienced a grade 4 acute hemorrhage from sphenopalatine artery during CIRT.^106^ The early age of the first treatment with X-RT of the analyzed data should prompt the multidisciplinary team to carefully consider how to better select patients that might be referred to high-tech RT at first diagnosis. In particular, attention should be given to identifying patients who could benefit from particle RT, and specifically with active scanning.^13^ In addition, various situations should be considered in our modern RT era in order to offer the best-tailored approach to our patients. The anamnestic conditions that can be related to abnormal radiosensitivity (ie, diabetes, obesity, autoimmune, and inflammatory disease), as well as genetic syndromes that are linked to higher intrinsic radiosensitivity (ie, ataxia-telangiectasia mutated, Fanconi anemia, Cockayne syndrome, Progeria syndrome, Neurofibromatosis)^3^, ^4^, ^5^, ^107^ should drive the decision-making. Considering the improvement in survival of oncological patients, the risk of developing a radio-induced cancer can not be underestimated and should always be discussed with the patient.

**Table 1:** An overview of the secondary tumors treated with particle beam radiation therapy.

Author	Sex	Age at primary diagnosis	Histology primary tumor	Site primary tumor (Stage)	Treatment primary tumor	Latency time	Histology secondary tumor	Site secondary tumor	Treatment	Particle radiation therapy treatment	Clinical outcome	Toxicity after particle radiation therapy
Barcellini et al (2021)^108^	F	69	Adenocarcinoma	Endometrium (Stage IB)	Surgery → RT+ BT	11 y	Squamous cell carcinoma	Vagina	Proton beam RT	IMPT: 39 GyE/13 fx	Alive with complete response at 1 y	Grade 1 vaginal dryness and atrophy
Romanello et al (2020)^109^	F	12	Hodgkin lymphoma	Neck (Stage III)	CT → RT	5 y	Low grade mucoepidermoid carcinoma	Parotid gland	Surgery + mixed beam particle RT	Mixed beam approach CIRT (6 GyE) followed by proton therapy (59.4 GyE to parotid bed, 50.4 GyE to perineural path	Alive and NED at 11 mo	Trismus G1 Xerostomia G1 Facial and trigeminal Nerve disorder G1
	M	3	Hodgkin lymphoma	Neck (stage IV)	CT → RT	31 y	Adenoid cystic carcinoma	Parotid gland	Surgery + CIRT	CIRT:68.8 GyE/16 fx	Alive (72 mo) with disease (lung metastases 2 y after surgery)	Trismus G1 Xerostomia G1 Facial and trigeminal Nerve disorder G1
Yang et al (2020)^110^	M	38^a^	Hodgkin lymphoma	Not provided	*Provided only the TD of RT* 50 Gy	24.5 y	Squamous cell carcinoma	Nasal-sinus	Surgery (R2)+ CIRT	CIRT: 60 GyE/20 fx	Alive and NED at 10.5 mo	Not deducible from the text
	M	55^a^	NPC	Not provided	*Provided only the TD of RT*: 69.7 Gy	5.58 y	Undifferentiated sarcoma	Nasal-sinus	Biopsy + Neoadjuvant and concurrent chemotherapy+CIRT	CIRT: 63GyE/23 fx	Alive and NED at 2.73 mo	Not deducible from the text
	M	34^a^	NPC	Not provided	Not provided	20 y	Myofibroblastic sarcoma	Nasopharynx	Surgery (R2)+CIRT	CIRT:60 GyE/20 fx	Alive and NED at 14.57 mo	Not deducible from the text
	F	37^a^	NPC	Not provided	*Provided only the TD of RT: 68 Gy*	14 y	Spindle cell sarcoma	Skull base	Biopsy+CIRT	CIRT: 69 GyE/23 fx	AWD at 13.87 mo	Not deducible from the text
	M	44^a^	NPC	Not provided	*Provided only the TD of RT: 68 Gy*	11 y	Pleomorphic sarcoma	Nasopharynx	Biopsy+CIRT	CIRT:60 GyE/20 fx	Died for toxicity at 5.97 mo	Acute: G4 hemorrhage from sphenopalatine artery Late: G5 hemorrhage
	M	55^a^	NPC	Not provided	*Provided only the TD of RT: 70.4 Gy*	5.3 y	Spindle cell sarcoma	Skull base	Biopsy + Neoadjuvant and concurrent chemotherapy+CIRT	CIRT: 69 GyE/23 fx	Alive and NED at 13 mo	Not deducible from the text
	M	56^a^	NPC	Not provided	Not provided	14 y	Osteosarcoma	Nasal-sinus	Biopsy+CIRT	CIRT:60 GyE/20 fx	Died for local disease at 8.1 mo	Not deducible from the text
	M	34^a^	Pituitary adenoma	Not provided	*Provided only the TD of RT: 13 Gy*	17.3 y	Spindle cell sarcoma	Skull base	Surgery (R2)+CIRT	CIRT:69 GyE/23 fx	Alive and NED at 8.8 mo	Not deducible from the text
	M	38^a^	NPC	Not provided	*Provided only the TD of RT: 72.25 Gy*	14.5 y	Sarcomatoid carcinoma	Nasal-sinus	Biopsy+CIRT	CIRT:63 GyE/21 fx	Died for local disease at 9.6 mo	Not deducible from the text
	M	40^a^	NPC	Not provided	*Provided only the TD of RT:86 Gy*	14.1 y	Olfactory neuroblastoma	Nasopharynx	Biopsy+Neoadjuvant chemotherapy+CIRT	CIRT:63 GyE/21 fx	Died for distant metastasis at 24.8 mo	Not deducible from the text
	F	21^a^	NPC	Not provided	*Provided only the TD of RT: 75.35 Gy*	11 y	Chondrosarcoma	Skull base	Surgery (R2)+CIRT	CIRT: 60 GyE/20 fx	Died for local disease at 11.67 mo	Not deducible from the text
	F	46^a^	Pituitary adenoma	Not provided	*Provided only the TD of RT:13 Gy*	10 y	Small round cell sarcoma	Skull base	Surgery (R2)+CIRT	CIRT: 60 GyE/20 fx	Died for local disease at 13.1 mo	Not deducible from the text
	M	50^a^	Myofibroblastic sarcoma	Not provided	*Provided only the TD of RT:57.6 Gy*	5.58 y	Myofibroblastic osteosarcoma	Nasal-sinus	Neoadjuvant chemotherapy+Surgery (R2)+CIRT	CIRT: 63 GyE/21 fx	Alive and NED at 18.46 mo	Not deducible from the text
	F	27^a^	Breast cancer	Not provided	*Provided only the TD of RT:60 Gy*	9 y	Angiosarcoma	Neck	Surgery (R0)	CIRT: 60 GyE/20 fx	Alive and NED at 27.57 mo	Not deducible from the text
	M	33^a^	Chordoma	Not provided	*Provided only the TD of RT: 70 Gy*	11.1 y	Chondrosarcoma	Skull base	Surgery (R2)	CIRT: 57.5 GyE/23 fx	Died for local disease at 29.63 mo	Not deducible from the text
Hayashi et al (2018)^111^	One Case	Not deducible from the text	Thymoma	Not deducible from the text	Not deducible from the text	10 y	Not deducible from the text	Lung	CIRT	Not deducible from the text	Not deducible from the text	Not deducible from the text
Guttmann et al (2017)^112^	Six Cases	Not deducible from the text	Non-sarcoma primary tumors	Not deducible from the text	Not deducible from the text	Not deducible from the text	Sarcomas (details are not deducible from the text)	Not deducible from the text	Proton beam RT	Not deducible from the text	Not deducible from the text	Acute: G3 dysphagia in 1 pt retreated to the neck for a sarcoma after a squamous cell cancer 11 y prior Not other details are deducible from the text
McDonald et al (2016)^113^	Six cases	Not deducible from the text	Non-sarcoma primary tumors	Not deducible from the text	Not deducible from the text	Not deducible from the text	Squamous cell carcinoma (4 pts) Non-squamous cell carcinoma (2 pts)	Head and neck	Proton beam RT	Not deducible from the text	Non-sarcoma primary tumors Higher incidence of distant metastases compared to recurrent tumor (66.7% vs 29.1%, *P*: .008)	Not deducible from the text

**Abbreviations:** RT, radiation therapy; BT, brachytherapy; CIRT, carbon ion radiotherapy; IMPT, intensity modulated proton therapy.

a Calculated considering the latency interval provided by the authors.

## Discussions

RT remains a crucial component of anticancer treatment, with more than 50% of patients undergoing RT during their oncological path. However, the primary obstacle that affects RT's effectiveness and ultimately contributes to treatment failure is the tumor's intrinsic radioresistance. This highlights the urgent need for an adequate selection of patients most likely to respond better to HT than to X-RT and, first of all, a thorough comprehension of the underlying radiobiology to effectively use these advantages to improve patient care.

Indeed, as for X-RT, the HT efficacy depends on various biological factors at subcellular, cellular, and microenvironmental levels: DNA damage "Repair" mechanisms induced by radiation, cell cycle "Redistribution," tumor cell "Repopulation," "Reoxygenation" of cells surviving radiation, inherent "Radiosensitivity," and the recently acknowledged "Reactivation" of the immune system's antitumor response.^114^ In this scenario, it is important to emphasize that, while PBT offers significant dosimetric advantages over conventional X-RT, it is less effective radiobiologically compared to heavier ions, such as carbon ion. However, hypofractionated PBT, which delivers higher doses per fraction over fewer treatment sessions, may help overcome the limitations of protons' lower RBE. The precise energy deposition and sharp dose gradient characteristic of protons make hypofractionation both feasible and effective, as it enables more accurate delivery of escalated doses to the tumor while minimizing damage to surrounding healthy tissues.

Today's RT is undergoing a significant revolution that extends far beyond the domains of physics and planning, with a primary focus on the comprehension of the molecular and systemic mechanisms behind radiosensitivity and radioresistance. But in the HT setting, we have merely begun to scratch the surface of what is conceivable thus far.

As we have described herein, HT is advantageous compared to X-RT for hypoxic, and slow-growing tumors by causing greater DNA damage, acting independently of the oxygen effect and the cell cycle phase. Moreover, recent literature has described the higher impact of HT on reactivating the antitumor immune response compared to X-RT.^10^, ^115^ All these radiobiological features should be considered during the tumor multidisciplinary board discussion to properly select the best approach and to ensure highly personalized oncological care that ethically should be both tumor and patient-driven.

However, for further advancements, a democratization of HT is needed, beginning with a framework for simpler and faster international access to particle facilities, to a reduction of costs passing through more and more solid interdisciplinary collaborations.

Indeed, while the number of PBT facilities is increasing worldwide, CIRT is currently only available at a limited number of hubs around the world, which makes it difficult for many patients to access this treatment. Moreover, HT is a relatively expensive form of RT, and its reimbursement is reliant on the nation's insurance system, limiting its use in certain health care settings.

In addition, there are limited long-term data on the efficacy and safety of CIRT, particularly in rare and radioresistant tumors. Preclinical investigations and clinical research must be conducted to delve into the mechanisms of tumor radioresistance and how these mechanisms can be intercepted by CIRT. The personalization of CIRT plans can be refined by focusing on RBE and LET integrations based on hypoxia or other radioresistant features. More recently, integrating multiion therapy can offer potential solutions to heterogeneous tumors, optimizing therapeutic outcomes in order to create more solid evidence. Future research should focus on expanding access to CIRT, combining CIRT with the most advanced systemic therapies (such as ICIs or synthetic lethal targeted therapy drugs). To make these steps forward, strong international collaborations between clinicians, physicists, and biologists are of pivotal importance. Furthermore, to effectively democratize the HT, we believe that the improvement of international cooperation in terms of knowledge sharing, education through scholarships for clinicians and health care managers, exchange programs, e-resources, free mentoring via technology, and public awareness campaigns can be strategic measures.

## Conclusions

Thanks to the emerging preclinical and clinical pieces of evidence, it is an exciting time for HT that appears as a promising treatment option for rare, radioresistant, recurrent, and radio-induced tumors, with potential benefits in terms of higher rates of LC and minimal toxicity. While there are several limitations associated with HT, especially CIRT, the available data suggest that it has the potential to transform the treatment and prognosis of the most challenging type of tumors. Further research is needed to fully understand the molecular underpinnings of HT and to identify ways to make HT more widely available to patients.

## Author Contributions

Ester Orlandi: Conceptualization. Barbara Vischioni and Amelia Barcellini: Methodology, Data curation, Writing- Original draft. Ester Orlandi, Juliette Thariat, and Marco Durante: Supervision, Review and Editing. Giuseppe Magro, Marco Rotondi, and Angelica Facoetti: Review and Editing.

## Declaration of Conflicts of Interest

Prof. Marco Durante, PhD, and Prof. Ester Orlandi, MD, are Associate Editors of the *International Journal of Particle Therapy.* The authors declare that they have no additional known competing financial interests or personal relationships that could have appeared to influence the work reported in this paper.
